# Body weight-supported gait training for patients with spinal cord injury: a network meta-analysis of randomised controlled trials

**DOI:** 10.1038/s41598-022-23873-8

**Published:** 2022-11-10

**Authors:** Fu-An Yang, Shih-Ching Chen, Jing-Fang Chiu, Ya-Chu Shih, Tsan-Hon Liou, Reuben Escorpizo, Hung-Chou Chen

**Affiliations:** 1 School of Medicine, College of Medicine, Taipei Medical University, Taipei, Taiwan; 2 Taiwan Society of Neurorehabilitation, Taipei, Taiwan; 3 Department of Physical Medicine and Rehabilitation, School of Medicine, College of Medicine, Taipei Medical University, Taipei, Taiwan; 4 Department of Physical Medicine and Rehabilitation, Taipei Medical University Hospital, Taipei, Taiwan; 5 Department of Physical Medicine and Rehabilitation, Shuang Ho Hospital, Taipei Medical University, No. 291 Zhongjheng Road, Zhonghe District, New Taipei City, 235 Taiwan; 6 Department of Rehabilitation and Movement Science, University of Vermont, College of Nursing and Health Sciences, Burlington, VT USA; 7 Swiss Paraplegic Research, Nottwil, Switzerland

**Keywords:** Neurology, Engineering

## Abstract

Different body weight-supported gait-training strategies are available for improving ambulation in individuals with spinal cord injury (SCI). These include body weight-supported overground training (BWSOGT), body weight-supported treadmill training (BWSTT), and robot-assisted gait training (RAGT). We conducted a network meta-analysis of randomised controlled trials (RCTs) to assess the effect and priority of each training protocol. We searched the PubMed, Cochrane Library, Scopus, and Embase databases from inception to 6 August 2022. The eligibility criteria were as follows: (1) being RCTs, (2) recruiting participants with SCI diagnosis and requiring gait training, (3) comparing different body weight-supported gait training strategies, and (4) involving ambulatory assessments. We conducted a network meta-analysis to compare different training strategies using the standard mean difference and its 95% credible interval. To rank the efficacy of training strategies, we used the P score as an indicator. Inconsistency in network meta-analysis was evaluated using loop-specific heterogeneity. We included 15 RCTs in this analysis. RAGT was had significantly more favourable performance than had the control intervention. The ranking probabilities indicated that the most effective approach was RAGT, followed by BWSOGT, BWSTT, and the control intervention. No significant inconsistency was noted between the results of the direct and indirect comparisons.

## Introduction

Following the acute phase of spinal cord injury (SCI), patients and their families must take on challenges such as restoring arm and hand function, regaining sexual function, improving bladder and bowel function, and enhancing walking ability^1–3^. Failure to restore ambulation before subjecting the patient to alternative gait training strategies leads to severe disability and psychosocial and economic problems^1,4^. One main strategy for the rehabilitation of patients with SCI is improving lower limb motor function^5,6^. Repetitive and intensive exercises can induce plasticity in the involved motor centres^7^. However, severe motor impairment in patients with SCI leads to fatigue, making it difficult for such patients to perform related exercises for a long period. Fatigue is thus a crucial limiting factor in conventional rehabilitation programmes^7^.

Body weight-supported training while walking is used in neurological rehabilitation^8–10^. It partially decreases the burden of load bearing and enables those who cannot walk to complete training protocols^11^. Body weight-supported overground training (BWSOGT) and body weight-supported treadmill training (BWSTT) are alternative training strategies for patients with SCI^12,13^. Automatic electromechanical devices are being increasingly used in neurorehabilitation^14,15^. Robot-assisted gait training (RAGT) has many advantages, such as maintenance of a physiological gait pattern and increases in training intensity and overall training duration^16–18^.

Although these train alternative training protocols—BWSOGT, BWSTT and RAGT—have been reported to be more favourable than conventional training, no study has compared them together. Therefore, we used a network meta-analysis approach for comparing the effectiveness of the three strategies for ambulatory improvements in patients with SCI. We conducted a comprehensive literature review to identify randomised controlled trials (RCTs) focusing on gait training for SCI.

## Methods

This network meta-analysis was registered prospectively in the International Prospective Register of Systematic Reviews database under the number CRD42021270919 on 29 August 2021. Our protocol adheres to the Preferred Reporting Items for Systematic Reviews and Meta-Analyses (PRISMA) extension statement for network meta-analysis^19^.

### Eligibility criteria

The eligibility criteria for studies were as follows: (1) being RCTs; (2) recruiting participants with an SCI diagnosis; (3) having an intervention group with different body weight-supported gait training strategies (RAGT, BWSTT, and BWSOGT); (4) having a control group with conventional gait training, such as sit to stand, static and dynamic standing balance, weight shifting, walking, turning, and stand to sit; and (5) involving ambulatory assessments. We excluded studies that (1) were not peer reviewed, such as conference papers and letters to the editor; (2) only presented protocols; (3) did not involve ambulatory assessments; and (4) used combination therapy. No language restrictions were applied.

### Search strategy

We independently reviewed the literature, extracted data, and performed crosschecks following the PRISMA guidelines (Supplementary Information)^20^. We searched the PubMed, Cochrane Library, Scopus, and Embase databases for relevant articles from inception to 6 August 2022 by using the following search string: ((spinal cord) OR (SCI) OR (myelopathy) OR (myelitis)) AND (((robot*) OR (RAGT) OR (end effector) OR (exoskeleton) OR (lokomat*) OR (locomat*)) OR (((locomot*) OR (treadmill)) AND (support*))). An additional search with other brand names of different body weight-supported gait training strategies was also conducted to facilitate a more detailed search. The following were used as keywords: Rysen, the Float, ZeroG, Keeogo, Dermoskeleton, ReWalk, Ekso Indego, HAL, WPAL, H2, REX, Ekso, ReWalk, Robin, CUHK-EXO, ITRI, Vanderbilt Exoskeleton, ARKE, Curara, Arazpour2103a, Kim2013, Chang2017, SMA, Kinesis, Lerner2017, Alter G Bionic Leg, Arazpour2013b, Kawasaki2017, Yeung2017, Boes2017, Welwalk, LiteGait, ALEX, LOPES, Gait Trainer, and Haptic Walker. RCTs were identified using the filter function of the databases. Additional articles were identified through a manual search of the reference lists of relevant articles. Two reviewers independently reviewed the full text of all potentially relevant articles to identify those that met the eligibility criteria. Any disagreement was resolved by a third reviewer.

### Study selection

After studies were retrieved from the databases, duplicate entries were removed using manual screening. Subsequently, the titles and abstracts of the remaining studies were screened so that relevant articles could be independently selected by two reviewers. Disagreements were resolved through mutual discussion or adjudication by a third reviewer. Subsequently, the full texts of the remaining articles were read in detail to determine the eligibility of the articles.

### Data items

The following data were obtained from each RCT: RCT type, American Spinal Injury Association Impairment Scale (AIS) grade, number and mean age of participants, protocol used in different groups, treatment duration, and outcome measurements.

### Outcome measurements

Ambulatory function impairment may limit daily activities and social performance. Thus, our primary outcome was walking ability. When data on walking ability were unavailable, another outcome measurement associated with ambulatory function was selected. The first priority was given to the 6-min walk test, which is recommended for the assessment of walking in patients with SCI^21^. The second priority was given to the 10-m walk test, which is also a recommended ambulation assessment method^21^. We determined the priority of the 6-min walk test prior to the 10-m walk test because although it is much easier to perform the 10-m walk test, the 6-min walk test is longer and therefore provides much more discriminative data on participant’s ambulation ability. The third priority was given to the lower extremity motor score, which is a standard neurologic assessment developed by the American Spinal Injury Association in which the voluntary muscle strength of five key muscle groups (hip flexors, knee extensors, ankle dorsiflexors, long toe extensors, and ankle plantarflexors) of both lower extremities are tested^22^. The fourth priority was given to the Walking Index for Spinal Cord Injury, which is an ordinal scale that evaluates the extent and nature of assistance (orthoses, supporting equipment such as walkers, and human helpers) that people with SCI require to be able to walk^23^. Only data on the highest ranking priority of ambulatory measures in each study were extracted for the network meta-analysis. Studies without ambulatory measurements were excluded. Data representing the longest duration of follow-up were pooled in the network meta-analysis.

### Risk-of-bias assessment

The risk of bias was assessed using the Cochrane RoB 2 tool, which is widely used for assessing the quality of RCTs^24^. We considered the overall bias and all five domains of bias: (1) bias arising from the randomisation process, (2) bias due to deviations from intended interventions, (3) bias due to missing outcome data, (4) bias in outcome measurements, and (5) bias in the selection of reported results^24^. In accordance with the Cochrane Handbook for Systematic Reviews of Interventions, the risk of bias was assessed by two independent reviewers^25^, and disagreements were resolved through discussion and consultation with a third reviewer.

### Statistical analysis

Network meta-analysis is a technique used to compare three or more interventions simultaneously in a single analysis through the combination of both direct and indirect evidence across a network of studies^25^. Network meta-analysis generates estimates of the relative effects between any pair of interventions in a network, and it usually yields estimates that are more precise than are single direct or indirect estimates^25^. It also allows the estimation of the ranking and hierarchy of interventions^25^. The network meta-analysis was performed using the ShinyNMA Version 1.01 website^26^ (https://jerryljw.shinyapps.io/ShinyNMA_/). This is a free online cloud computing network meta-analysis website for researchers, and it can be used to create charts as per the standards of the latest PRISMA 2020 guidelines^20^. It synthesises results and provides a rationale for choosing R software (version 4.1.0) and specific packages, namely metafor (version 2.4-0), netmeta (version 1.3-0), or BUGSnet (version 1.0-4).

We extracted continuous data by changing the baseline measurements. In the absence of standard deviation values, data were estimated through the calculation of correlation coefficients in accordance with the instructions in the Cochrane Handbook for Systematic Reviews of Interventions^25^. The transitivity assumption underlying network meta-analysis was evaluated by comparing the distribution of clinical and methodological variables that could serve as effect modifiers across treatment comparisons^25^. A random-effects model was used in this network meta-analysis. We conducted a head-to-head comparison of body weight-supported gait training for SCI by estimating the standard mean difference (SMD) and 95% credible interval (CI). Furthermore, we analysed the distribution of probabilities in the ranking of body weight-supported gait training strategies for ambulatory improvement among patients with SCI. For efficacy ranking, we used the P score as an indicator. The P score is used to measure the extent of certainty that a treatment is better than other treatments, averaged over all competing treatments^27^. It is rated from 0 (worst) to 1 (best). If one treatment is better than the other treatments, its P score is higher than that for other treatments. Moreover, inconsistency in network meta-analysis was evaluated using loop-specific heterogeneity and local incoherence estimates and by comparing differences in effect sizes between standard meta-analyses (direct comparisons) and indirect comparisons^25^.

## Results

### Study selection

**Figure 1:**
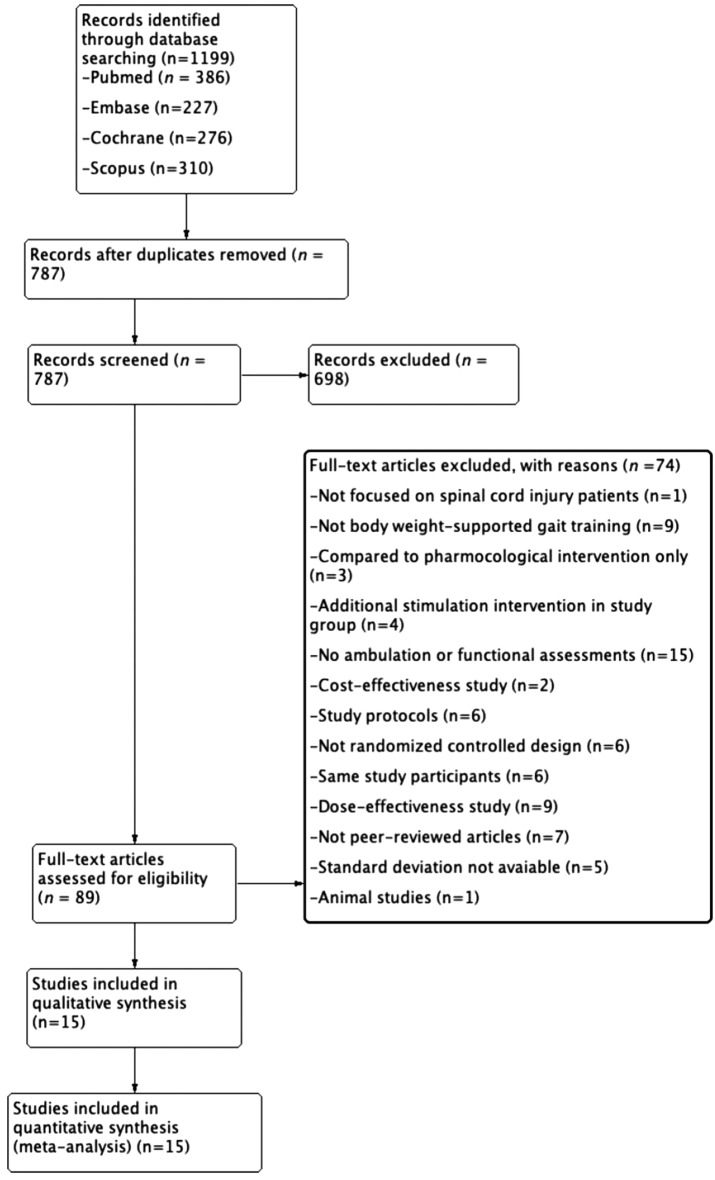
Flowchart of study selection.

We initially retrieved 1199 RCTs and excluded 412 duplicates. After title and abstract screening, 698 studies were excluded. The full texts of the remaining 89 papers were screened; among them, 1 study did not focus on patients with SCI, 9 did not involve body weight-supported ambulation training, 3 compared only pharmacological interventions, 4 included additional stimulation in the study group, 15 did not include ambulation or functional assessment, 2 were cost-effectiveness studies, 6 were study protocols, 6 were not RCTs, 6 had the same study group, 9 were dose-effectiveness studies, 7 were not peer-reviewed articles, 5 did not provide standard deviations, and 1 was an animal study. Finally, 15 articles were selected for this network meta-analysis^7,28–41^ (Fig. 1).

### Characteristics of the included studies

In the 15 selected RCTs,three body weight-supported gait training protocols were used, namely RAGT^7,28,30,31,33,34,36–41^, BWSTT^29,32,35,37^, and BWSOGT^29,35^. Conventional gait training was prespecified as a control intervention. Alexeeva et al. conducted a three-arm study comparing BWSTT, BWSOGT, and a control intervention^29^. Labruyère et al. conducted a crossover RCT^33^. According to the Cochrane Handbook for Systematic Reviews of Interventions, the inclusion of crossover studies in a network meta-analysis is acceptable^25^. In addition, including the final outcome data is more appropriate than including only the outcome data from the first period (before the crossover). We followed these guidelines in our network meta-analysis. Table 1 summarises the main characteristics of the 15 RCTs.

**Table 1:** Characteristics of the selected randomised controlled trials.

Author, year	RCT type	Group	AIS grade	Protocol	n	Time after injury (year), mean (SD)	Age (year), mean (SD)	Treatment duration	Outcome measurements
Alexeeva et al., 2011^29^	Parallel RCT	1	C-D	Control intervention	12	8.04 (7.4)	37.3 (12.99)	13 weeks	10MWT^a^
		2	C-D	BWSTT	9	4.5 (3.6)	43.3 (15.76)		
		3	C-D	BWSOGT	14	7.9 (9.7)	36.4 (12.87)		
Cheung et al., 2019^30^	Parallel RCT	1	C-D	RAGT	8	17 (7.0) months	55.6 (4.98)	8 weeks	LEMS^a^ and WISCI
		2	C-D	Control intervention	8	10.4 (6.3) months	53.0 (12.94)		
Field-Fote et al., 2011^32^	Parallel RCT	1	C-D	BWSTT	17	> 1 year	39.3 (14.6)	12 weeks	LEMS^a^
		2	C-D	RAGT	14		45 (8.0)		
Labruyère et al., 2014^33^	Crossover RCT	1	C-D	RAGT	5	39.6 (27.3) months	58.8 (11.0995)	8 weeks	10MWT^a^, LEMS, and WISCI
		2	C-D	Control intervention	4	63.2 (83.9) months	59.25 (12.7639)		
Mıdık et al., 2020^34^	Parallel RCT	1	C-D	RAGT	15	5 (4–30) (median (interquartile range))	35.4 (12.1)	5 weeks	LEMS^a^ and WISCI
		2	C-D	Control intervention	15	24 (17–44) (median (interquartile range))	37.9 (10.0)		
Senthilvelkumar et al., 2015^35^	Parallel RCT	1	C	BWSOGT	7	5.9 (4.7) months	36.5 (13.8)	8 weeks	LEMS^a^ and WISCI
		2	C	BWSTT	7	5.9 (5.2) months	33.8 (13.6)		
Wu et al., 2018^37^	Parallel RCT	1	C-D	RAGT	7	5.8 (2.8)	48.4 (13.5)	6 weeks	6MWT^a^ and LEMS
		2	C-D	BWSTT	7	9.4 (8.4)	48.1 (4.9)		
Esclar ´ın-Ruz et al., 2014^31^	Parallel RCT	1	C-D	RAGT	21	125.6 (65.2) days	43.6 (12)	8 weeks	6MWT^a^, 10MWT, LEMS, and WISCI
		2	C-D	Control intervention	21	140.3 (45.5) days	44.9 (7)		
Lin et al., 2016^38^	Parallel RCT	1	C-D	RAGT	8	3.25 (0.93) months	44.00 (6.02)	12 weeks	LEMS^a^ and WISCI
		2	C-D	Control intervention	8	3.19 (1.22) months	47.50 (5.53)		
Alcobendas-Maestro et al., 2012^28^	Parallel RCT	1	C-D	RAGT	37	120 (87.5–145) days (median (interquartile range))	45.2 (15.5)	8 weeks	6MWT^a^, 10MWT, LEMS and WISCI
		2	C-D	Control intervention	38	135 (93.7–180) days (median (interquartile range))	49.5 (12.8)		
Shin et al., 2014^36^	Parallel RCT	1	D	RAGT	27	3.33 (2.02) months	43.15 (14.37)	4 weeks	LEMS^a^
		2	D	Control intervention	26	2.73 (1.97) months	48.15 (11.49)		
Yildirim et al., 2019^7^	Parallel RCT	1	A-D	RAGT	44	3 (2) months	32 (23)	8 weeks	WISCI^a^
		2	A-D	Control intervention	44	3 (2) months	36.5 (24)		
Edwards et al., 2022^39^	Parallel RCT	1	C-D	RAGT	9	8.4 (2.45)	42.8 (7.2)	12 weeks	10MWT^a^ and WISCI
		2	C-D	Control intervention	10	7.3 (1.56)	47.1 (8.3)		
Piira et al., 2020^40^	Parallel RCT	1	C-D	RAGT	16	14.6 (17.2)	50 (13)	24 weeks	6MWT^a^, 10MWT, and LEMS
		2	C-D	Control intervention	21	11.1 (15.0)	49 (14)		
Xiang et al., 2021^41^	Parallel RCT	1	A-C	RAGT	9	2 (4.5) months	39.8 (12.2)	4 weeks	6MWT^a^ and LEMS
		2	A-C	Control intervention	9	2 (0.5) months	36.6 (11.8)		

^a^Outcome selected in this network meta-analysis.

RCT, randomised controlled trial; AIS, American Spinal Injury Association Impairment Scale; SD, standard deviation; BWSTT, body weight-supported treadmill training; BWSOGT, body weight–supported overground training; RAGT, robot-assisted gait training; 10MWT, 10-m walk test; 6MWT, 6-min walk test; LEMS, lower extremity motor score; WISCI, Walking Index for Spinal Cord Injury.

### Risk-of-bias assessment

**Figure 2:**
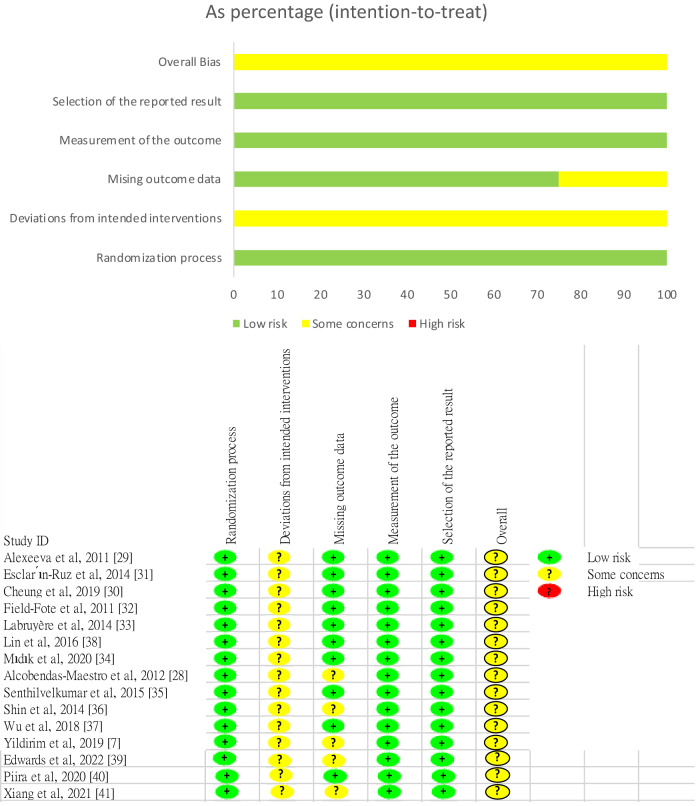
Study quality assessment.

Two reviewers assessed the quality of the selected RCTs by using the RoB 2 tool^24^. All studies had a low risk of bias in terms of the randomisation process^7,28–41^. Some concerns were noted for all studies in terms of deviation from the intended intervention^7,28–41^. Five studies had some concerns regarding missing outcome data^7,28,36,39,41^, and a low risk of bias was noted for 10 studies^29–35,37,38,40^. All studies exhibited a low risk of bias in outcome measurement^7,28–41^. All studies exhibited a low risk of bias in the selection of reported results^7,28–41^. The overall risk of bias was uncertain for all studies^7,28–41^ (Fig. 2).

### Synthesis of results: network meta-analysis

**Figure 3:**
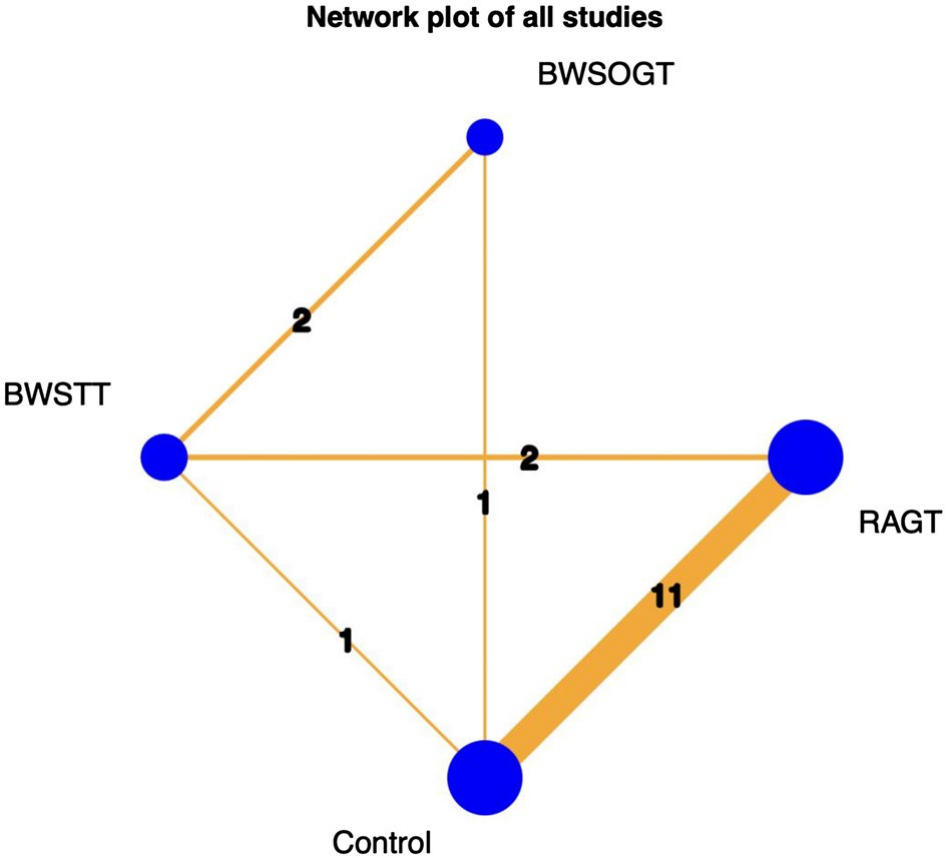
Network plot of all studies. The nodes, which represent the interventions in the network, and the lines, which highlight the available direct comparisons between pairs of interventions. The size of the nodes and the width of the lines both represent the number of studies. RAGT, robot-assisted gait training; BWSTT, body weight-supported treadmill training; BWSOGT, body weight-supported overground training.

**Figure 4:**
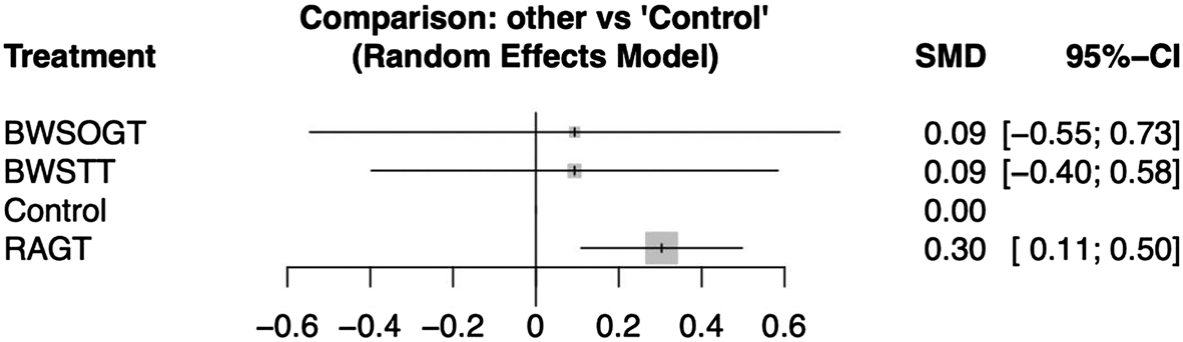
Forest plot of ambulatory assessments. The SMDs and 95% CIs of comparison between the control intervention and other body weight-supported gait training therapies were as follows: RAGT = 0.30 (0.11, 0.50); BWSTT = 0.09 (− 0.40, 0.58); and BWSOGT = 0.09 (− 0.55, 0.73). RAGT, robot-assisted gait training; BWSTT, body weight-supported treadmill training; BWSOGT, body weight-supported overground training; SMD, standard mean difference; 95% CI, 95% credible interval.

**Figure 5:**
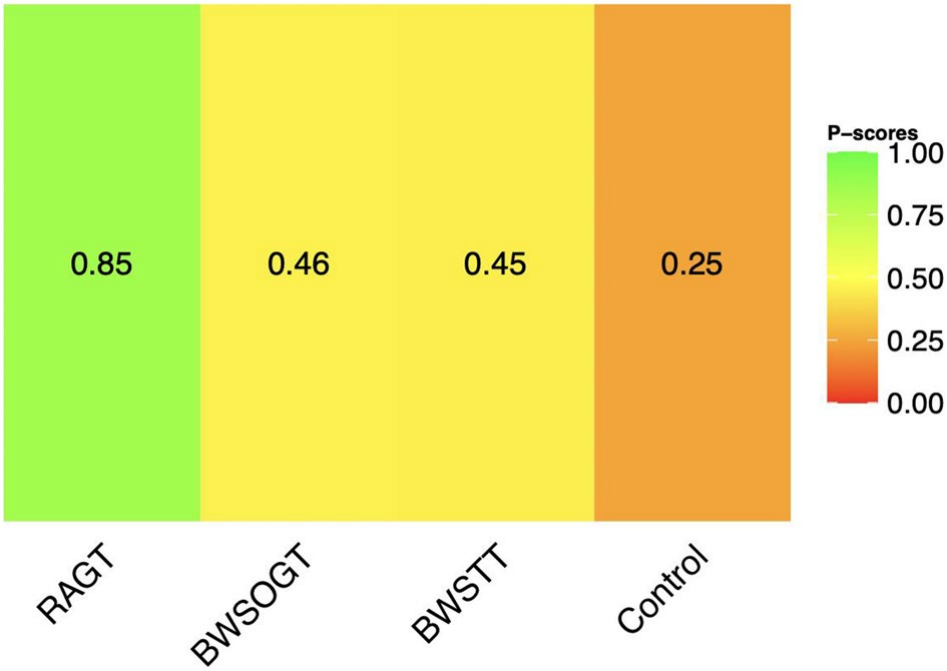
Distribution of probabilities in the ranking of each body weight–supported gait training strategy. The ranking probabilities indicated that RAGT was the most effective, followed by BWSOGT, BWSTT, and the control intervention. RAGT, robot-assisted gait training; BWSTT, body weight-supported treadmill training; BWSOGT, body weight–supported overground training.

Our network meta-analysis included 497 participants across 15 RCTs. Figure 3 presents a network diagram of the included body weight-supported gait training therapies. At least one placebo-controlled trial was included for each therapy. The pooled SMDs of functional scores in the network meta-analysis revealed that RAGT was significantly more favourable than the control intervention, whereas BWSTT and BWSOGT did not result in significant differences compared with the control intervention. The SMDs and 95% CIs from comparisons between the control intervention and other body weight-supported gait training therapies were as follows: RAGT = 0.30 (0.11, 0.50), BWSTT = 0.09 (− 0.40, 0.58), and BWSOGT = 0.09 (− 0.55, 0.73; Fig. 4). Moreover, we synthesised head-to-head studies separately to assess differences among body weight-supported gait training strategies. Table 2 presents the results of the pairwise meta-analysis and network meta-analysis of walking ability with overall training. Furthermore, the distribution of probabilities in the ranking of each training strategy was analysed. The ranking probabilities indicated that RAGT was the most effective, followed by BWSOGT, BWSTT, and the control intervention (Fig. 5).

**Table 2:** Network meta-analysis results related to functional scores.

Pairwise meta-analysis			
**RAGT**	–	0.11 [− 0.47; 0.70]	0.31 [0.12; 0.51]
0.21 [− 0.44; 0.86]	**BWSOGT**	0.07 [− 0.58; 0.73]	− 0.01 [− 0.79; 0.76]
0.21 [− 0.27; 0.69]	0.00 [− 0.60; 0.60]	**BWSTT**	− 0.09 [− 0.95; 0.78]
0.30 [ 0.11; 0.50]	0.09 [− 0.55; 0.73]	0.09 [− 0.40; 0.58]	**Control intervention**
Network meta-analysis			

Data are expressed as standard mean differences [95% credible interval (network meta-analysis; 95% confidence interval (pairwise meta-analysis)]. Significant results are underlined. “–” indicates data are not applicable.

The lower triangle represents the network meta-analysis results, and the upper triangle represents the pairwise meta-analysis results.

RAGT, robot-assisted gait training; BWSTT, body weight-supported treadmill training; BWSOGT, body weight-supported overground training.

### Network consistency

Network plots contain nodes, which represent the interventions in the network, and lines, which highlight the available direct comparisons between pairs of interventions^25^. The size of nodes and the width of lines both represent the number of studies. Our network plot (Fig. 3) depicts two triangle loops (RAGT-BWSTT-control intervention and BWSTT-BWSOGT-control intervention), and the loop-specific heterogeneity revealed no significant inconsistency between the results of direct and indirect comparisons (Table 3).

**Table 3:** Assessment of inconsistency among the included studies.

Comparison	Number of studies	Network meta-analysis	Direct comparison	Indirect comparison	Difference between direct and indirect comparison	Lower limit of 95% CI	Upper limit of 95% CI	P-value
BWSOGT: BWSTT	2	0.0002	0.07	− 0.4	0.47	− 1.19	2.14	0.58
BWSOGT: control intervention	1	0.09	− 0.01	0.33	− 0.35	− 1.72	1.03	0.62
BWSTT: control intervention	1	0.09	− 0.09	0.18	− 0.27	− 1.32	0.78	0.62
BWSTT: RAGT	2	− 0.21	− 0.11	− 0.4	0.29	− 0.73	1.30	0.58
RAGT: control intervention	11	0.30	0.31	0.027	0.29	− 0.73	1.30	0.58

CI, credible interval; RAGT, robot-assisted gait training; BWSTT, body weight-supported treadmill training; BWSOGT, body weight-supported overground training.

**Figure 6:**
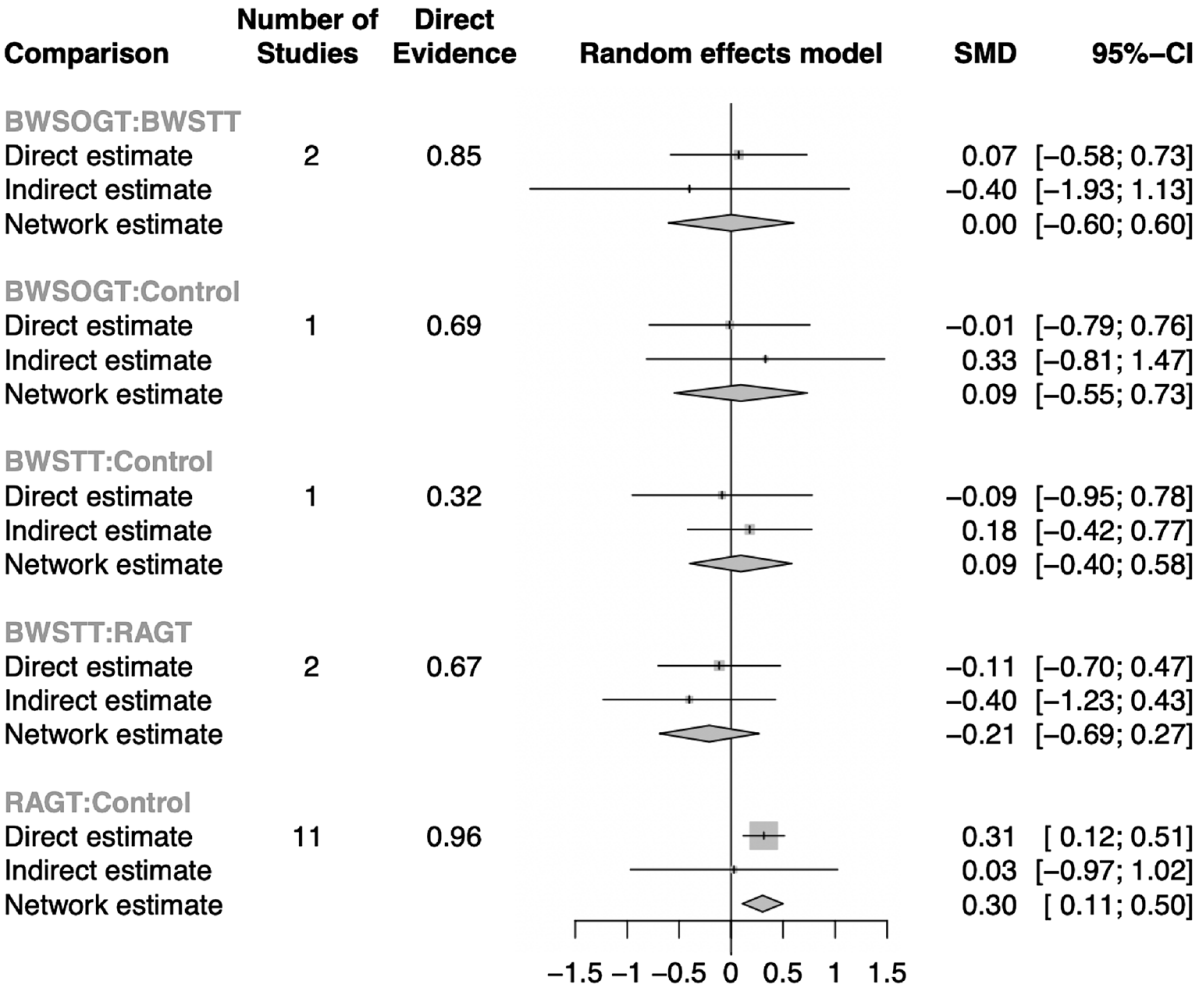
Forest plots of pairwise meta-analyses and network meta-analyses of ambulatory assessments. RAGT, robot-assisted gait training; BWSTT, body weight-supported treadmill training; BWSOGT, body weight-supported overground training.

Furthermore, the differences between the traditional pairwise meta-analyses and network meta-analyses were determined and are presented as forest plots (Fig. 6); none of the differences were significant.

### Adverse events

Of the 15 selected RCTs, six reported on adverse events^29,30,33,35,39,41^. No adverse events were observed in four studies^30,33,35,41^, and two reported that some participants had experienced pain^29,39^. The investigated interventions were relatively safe and well tolerated by participants.

## Discussion

Our results revealed that the body weight-supported gait training protocol with the highest ranking was RAGT followed by BWSOGT, BWSTT, and the control intervention. However, only RAGT was significantly more effective than the control intervention. No significant inconsistency was noted in our network. Moreover, our quality assessment results revealed that most of the included studies had an acceptable risk of bias.

In our network meta-analysis, RAGT ranked first as a body weight-supported gait training protocol for patients with SCI. According to a systematic review conducted by Antonio et al., many rehabilitation robots are available and can be classified as grounded exoskeletons, end-effector devices, wearable exoskeletons, and soft exoskeletons^42^. Although all these devices are robot assisted, some provide only guidance and gait modulation without body weight support. To ensure comparability with other body weight-supported gait training devices, we focused on body weight-supported grounded exoskeletons.

RAGT aims to improve the walking ability of patients with SCI. Our results supported its use by these patients. Robotic training has become readily accessible in rehabilitation centres, and robot-assisted gait rehabilitation has received much attention owing to its benefits for people with neurological conditions^30,43^. Robotic training may be an attractive option for patients and their families because of its sophistication and use of computer interface that offers a virtual reality experience both biofeedback^43^. This technology is also appealing to therapists because they potentially require fewer staff members and cause less physical strain than conventional therapy^44^. Robotic orthoses provide guidance on lower limb movement during walking training, facilitating prolonged walking training with the afferent input of a normal gait pattern^30^. This extensive exposure within task-specific repetitive training promotes the reorganisation of the primary motor cortex, and functional outcomes can be improved in patients with neurological conditions^45^.

An effective gait requires multifactorial system control, including control of the neuromuscular, musculoskeletal, cardiopulmonary, sensory, and cognitive systems. Robot-assisted devices may help improve neural plasticity—the tendency of synapses and neural circuits to change in response to activity—by providing intensive locomotor gait training^46,47^. Furthermore, intensive training helps prevent the age-related process of deconditioning, the onset and progression of impairment, functional limitation, disability, and changes in physical function and health resulting from injury, disease, and other causes^48^.

Body weight-supported gait training has been widely advocated for people with SCI and has been demonstrated to improve ambulatory ability^29,35,49,50^. It enables patients to walk with improved gait pattern while their weight bearing stress is relieved^35^. BWSOGT combines body weight-supported training and over ground training, whereas BWSTT is a combination of body weight-supported gait training and treadmill training. In BWSTT, walking speed is almost constant owing to the use of a treadmill belt, thereby emphasising the rhythmicity of voluntary movements^29^. Although these two training strategies may improve ambulatory ability, few studies have illustrated their significance. Several novel devices are available, such as Rysen, the Float, and ZeroG. However, no RCTs have evaluated gait training for SCI by focusing on these devices. Therefore, future high-quality RCTs, especially those focusing on novel devices, are required to evaluate their potential for improving ambulatory function in patients with SCI.

Several studies have illustrated the effectiveness of RAGT. Duan et al., through a pairwise meta-analysis, concluded that RAGT is more effective than conventional training in improving ambulation^51^. Furthermore, through a pairwise meta-analysis, Fang et al. reported that RAGT can improve spasticity and walking ability in people with SCI better than conventional training can^52^. In a systematic review of 13 RCTs, Mehrholz et al. compared different training strategies to improve gait in people with SCI and concluded that BWSTT and RAGT do not increase walking speed more than overground gait training and other forms of physiotherapy, but their effects on walking distance are unclear^53^. Although these studies have provided diverse outcomes on different training strategies for patients with SCI, they used only pairwise meta-analysis; these results lack indirect comparisons. Therefore, we performed both direct and indirect comparisons in the current network meta-analysis to investigate the efficacy of these interventions. Our network plot had two closed loops (Fig. 3). In a closed loop, each direct source of evidence is complemented by an indirect source of evidence for the same comparison^25^, thereby providing much more solid evidence than an open loop can. Thus, network meta-analyses have the advantage of estimating relative effects between any pair of interventions in the network, usually yielding more precise estimates than a single direct or indirect estimate.

The studies included in our analyses had high consistency and an acceptable risk of bias. This implies that the different sources of evidence (direct and indirect) agree with each other^25^. However, the transitivity of this study might be influenced by different characteristics among the participants and protocols of the selected RCTs. Regarding the AIS grade of the participants, one study included participants with AIS grade A^7^, whereas the others reported patients with grades C to D^28–40^ and one reported with grade A to C^41^. Furthermore, the treatment duration was different, ranging from 4 weeks^36,41^ to > 10 weeks^29,32,38–40^. These discrepancies may have affected the transitivity of this study.

This network meta-analysis has several strengths. First, this is the first network meta-analysis of RCTs that focused on the effect of different body weight-supported gait training approaches for patients with SCI. Second, no significant inconsistency was noted between the results of the direct trials and indirect comparisons, indicating favourable coherence. Third, multiple major databases were used to identify RCTs without language restrictions. Finally, the risk of bias of the selected RCTs was mostly acceptable.

This study also had several limitations. First, the number of included articles was relatively small, particularly those focused on BWSOGT and BWSTT, for conducting a network meta-analysis. This might make RAGT a dominant intervention in this study. Second, the transitivity of this study may have been influenced by the different treatment protocols and participant characteristics of the included studies. Thus, caution should be exercised when applying our results to other patient groups. Third, among the various robot-assisted devices available, we focused only on body weight-supported devices. To overcome these limitations, larger-scale studies focusing on BWSOGT or BWSTT are warranted to determine the effectiveness of these protocols. Moreover, future studies should attempt to include consistent protocols and participant characteristics.

## Conclusion

We conducted the first network meta-analysis of RCTs focusing on body weight-supported gait training for patients with SCI. Among them, RAGT was the most effective, followed by BWSOGT, BWSTT, and the control intervention. We suggest that RAGT should be the training protocol of choice for improving walking ability in individuals with SCI. Because few studies have focused on BWSOGT and BWSTT, future studies should comprehensively evaluate their potential for improving the walking ability of patients with SCI. Moreover, further high-quality, large-scale RCTs are required to ensure the benefits and long-term effects of these interventions.

## Supplementary Information

Supplementary Information.

## Data Availability

The datasets generated during and analyzed during the current study are available from the corresponding author on reasonable request.

## References

[1] Nas K, Yazmalar L, Şah V, Aydın A, Öneş K (2015). Rehabilitation of spinal cord injuries. World J. Orthop..

[2] Anderson KD (2004). Targeting recovery: Priorities of the spinal cord-injured population. J. Neurotrauma.

[3] Eng JJ (2007). Spinal cord injury rehabilitation evidence: Methods of the SCIRE systematic review. Top. Spinal Cord Inj. Rehabil..

[4] Harvey LA (2016). Physiotherapy rehabilitation for people with spinal cord injuries. J. Physiother..

[5] Fehlings MG, Cadotte DW, Fehlings LN (2011). A series of systematic reviews on the treatment of acute spinal cord injury: A foundation for best medical practice. J. Neurotrauma.

[6] Nasser MET, Reda MAEH, Awad MR, Amin IR, Assem SA (2014). Effect of massed practice and somatosensory stimulation on the upper extremity function in patients with incomplete cervical spinal cord injury. Alexandria J. Med..

[7] Yıldırım MA, Öneş K, Gökşenoğlu G (2019). Early term effects of robotic assisted gait training on ambulation and functional capacity in patients with spinal cord injury. Turk. J. Med. Sci..

[8] Kurz MJ, Stuberg W, Dejong S, Arpin DJ (2013). Overground body-weight-supported gait training for children and youth with neuromuscular impairments. Phys. Occup. Ther. Pediatr..

[9] Sousa CO, Barela JA, Prado-Medeiros CL, Salvini TF, Barela AM (2009). The use of body weight support on ground level: An alternative strategy for gait training of individuals with stroke. J. Neuroeng. Rehabil..

[10] Sousa CO, Barela JA, Prado-Medeiros CL, Salvini TF, Barela AM (2011). Gait training with partial body weight support during overground walking for individuals with chronic stroke: A pilot study. J. Neuroeng. Rehabil..

[11] Ada L, Dean CM, Vargas J, Ennis S (2010). Mechanically assisted walking with body weight support results in more independent walking than assisted overground walking in non-ambulatory patients early after stroke: A systematic review. J. Physiother..

[12] Wirz M (2005). Effectiveness of automated locomotor training in patients with chronic incomplete spinal cord injury: A multicenter trial. Arch. Phys. Med. Rehabil..

[13] Dobkin BH (1999). An overview of treadmill locomotor training with partial body weight support: A neurophysiologically sound approach whose time has come for randomized clinical trials. Neurorehabil. Neural Repair.

[14] Tefertiller C, Pharo B, Evans N, Winchester P (2011). Efficacy of rehabilitation robotics for walking training in neurological disorders: A review. J. Rehabil. Res. Dev..

[15] Dobkin BH, Duncan PW (2012). Should body weight-supported treadmill training and robotic-assistive steppers for locomotor training trot back to the starting gate?. Neurorehabil. Neural Repair.

[16] AuYong N, Lu DC (2014). Neuromodulation of the lumbar spinal locomotor circuit. Neurosurg. Clin. N. Am..

[17] Nam KY (2017). Robot-assisted gait training (Lokomat) improves walking function and activity in people with spinal cord injury: A systematic review. J. Neuroeng. Rehabil..

[18] Vaney C (2012). Robotic-assisted step training (lokomat) not superior to equal intensity of over-ground rehabilitation in patients with multiple sclerosis. Neurorehabil. Neural Repair.

[19] Hutton B (2015). The PRISMA extension statement for reporting of systematic reviews incorporating network meta-analyses of health care interventions: Checklist and explanations. Ann. Intern. Med..

[20] Page MJ (2021). The PRISMA 2020 statement: An updated guideline for reporting systematic reviews. BMJ.

[21] Scivoletto G (2011). Validity and reliability of the 10-m walk test and the 6-min walk test in spinal cord injury patients. Spinal Cord.

[22] Shin JC, Yoo JH, Jung TH, Goo HR (2011). Comparison of lower extremity motor score parameters for patients with motor incomplete spinal cord injury using gait parameters. Spinal Cord.

[23] Ditunno JF (2013). The Walking Index for Spinal Cord Injury (WISCI/WISCI II): Nature, metric properties, use and misuse. Spinal Cord.

[24] Higgins JPT (2011). The Cochrane Collaboration’s tool for assessing risk of bias in randomised trials. BMJ.

[25] Higgins JPT TJ CJ CM, L. T., Page MJ, Welch VA (editors). Cochrane Handbook for Systematic Reviews of Interventions version 6.2 (updated February 2021). Cochrane http://www.training.cochrane.org/handbook (2021).

[26] WEI-MING H, R.-W., LIOU., & SHEN-HUA, LIN. ShinyNMA. https://jerryljw.shinyapps.io/ShinyNMA_/ (Accessed 7 Aug 2021).

[27] Rücker G, Schwarzer G (2015). Ranking treatments in frequentist network meta-analysis works without resampling methods. BMC Med. Res. Methodol..

[28] Alcobendas-Maestro M (2012). Lokomat robotic-assisted versus overground training within 3 to 6 months of incomplete spinal cord lesion: Randomized controlled trial. Neurorehabil. Neural Repair.

[29] Alexeeva N (2011). Comparison of training methods to improve walking in persons with chronic spinal cord injury: A randomized clinical trial. J. Spinal Cord Med..

[30] Cheung EYY (2019). Effect of EMG-biofeedback robotic-assisted body weight supported treadmill training on walking ability and cardiopulmonary function on people with subacute spinal cord injuries—A randomized controlled trial. BMC Neurol..

[31] Esclarín-Ruz A (2014). A comparison of robotic walking therapy and conventional walking therapy in individuals with upper versus lower motor neuron lesions: A randomized controlled trial. Arch. Phys. Med. Rehabil..

[32] Field-Fote EC, Roach KE (2011). Influence of a locomotor training approach on walking speed and distance in people with chronic spinal cord injury: A randomized clinical trial. Phys. Ther..

[33] Labruyère R, van Hedel HJ (2014). Strength training versus robot-assisted gait training after incomplete spinal cord injury: A randomized pilot study in patients depending on walking assistance. J. Neuroeng. Rehabil..

[34] Mıdık M, Paker N, Buğdaycı D, Mıdık AC (2020). Effects of robot-assisted gait training on lower extremity strength, functional independence, and walking function in men with incomplete traumatic spinal cord injury. Turk. J. Phys. Med. Rehabil..

[35] Senthilvelkumar T, Magimairaj H, Fletcher J, Tharion G, George J (2015). Comparison of body weight-supported treadmill training versus body weight-supported overground training in people with incomplete tetraplegia: A pilot randomized trial. Clin. Rehabil..

[36] Shin JC, Kim JY, Park HK, Kim NY (2014). Effect of robotic-assisted gait training in patients with incomplete spinal cord injury. Ann. Rehabil. Med..

[37] Wu M, Kim J, Wei F (2018). Facilitating weight shifting during treadmill training improves walking function in humans with spinal cord injury: A randomized controlled pilot study. Am. J. Phys. Med. Rehabil..

[38] Lin HD, Zhang T, Chen Q (2016). Effect of robot-assisted gait training on walking ability in patients with incomplete spinal cord injury. Acta Automatica Sinica.

[39] Edwards DJ (2022). Walking improvement in chronic incomplete spinal cord injury with exoskeleton robotic training (WISE): A randomized controlled trial. Spinal Cord.

[40] Piira A (2020). Quality of life and psychological outcomes of body-weight supported locomotor training in spinal cord injured persons with long-standing incomplete lesions. Spinal Cord.

[41] Xiang XN (2021). Exoskeleton-assisted walking improves pulmonary function and walking parameters among individuals with spinal cord injury: A randomized controlled pilot study. J. Neuroeng. Rehabil..

[42] Rodríguez-Fernández A, Lobo-Prat J, Font-Llagunes JM (2021). Systematic review on wearable lower-limb exoskeletons for gait training in neuromuscular impairments. J. Neuroeng. Rehabil..

[43] Pool D, Valentine J, Taylor NF, Bear N, Elliott C (2021). Locomotor and robotic assistive gait training for children with cerebral palsy. Dev. Med. Child Neurol..

[44] Ammann-Reiffer C, Bastiaenen CH, Meyer-Heim AD, van Hedel HJ (2017). Effectiveness of robot-assisted gait training in children with cerebral palsy: A bicenter, pragmatic, randomized, cross-over trial (PeLoGAIT). BMC Pediatr..

[45] Ungerleider LG, Doyon J, Karni A (2002). Imaging brain plasticity during motor skill learning. Neurobiol. Learn. Mem..

[46] Wang HY, Yang YH (2006). Evaluating the responsiveness of 2 versions of the gross motor function measure for children with cerebral palsy. Arch. Phys. Med. Rehabil..

[47] Cauraugh JH, Summers JJ (2005). Neural plasticity and bilateral movements: A rehabilitation approach for chronic stroke. Prog. Neurobiol..

[48] Guide to Physical Therapist Practice (1997). Part 1: A description of patient/client management. Part 2: Preferred practice patterns. American Physical Therapy Association. Phys. Ther..

[49] Wirz M, Bastiaenen C, de Bie R, Dietz V (2011). Effectiveness of automated locomotor training in patients with acute incomplete spinal cord injury: A randomized controlled multicenter trial. BMC Neurol..

[50] Hicks AL (2005). Long-term body-weight-supported treadmill training and subsequent follow-up in persons with chronic SCI: Effects on functional walking ability and measures of subjective well-being. Spinal Cord.

[51] Duan R (2021). Clinical benefit of rehabilitation training in spinal cord injury: A systematic review and meta-analysis. Spine.

[52] Fang CY, Tsai JL, Li GS, Lien AS, Chang YJ (2020). Effects of robot-assisted gait training in individuals with spinal cord injury: A meta-analysis. Biomed. Res. Int..

[53] Mehrholz J, Harvey LA, Thomas S, Elsner B (2017). Is body-weight-supported treadmill training or robotic-assisted gait training superior to overground gait training and other forms of physiotherapy in people with spinal cord injury? A systematic review. Spinal Cord.

